# Supplementary material for: Tuberculosis dissemination in kidney transplant recipient treated with anti-CD40 monoclonal antibody: a case report
Source: BMC Nephrol. 2022 Aug 19;23:290. doi: 10.1186/s12882-022-02916-2 (PMC9388963; doi:10.1186/s12882-022-02916-2)
Supplement: Supplementary file 1 — Additional file 1. CARE Checklist of information to include when writing a case report. [file 12882_2022_2916_MOESM1_ESM.pdf]

CARE case report guidelines

CARE Checklist of information to include when writing a case report

(cc) BY-NC-ND

Topic	Item	Checklist item description	Reported on Line
Title	1	The diagnosis or intervention of primary focus followed by the words "case report"	P1, Title
Key Words	2	2 to 5 key words that identify diagnoses or interventions in this case report, including "case report"	P2, Key words
Abstract (no references)	3a	Introduction: What is unique about this case and what does it add to the scientific literature?	P2, Background, Paragr. 1
	3b	Main symptoms and/or important clinical findings	P2, Case present, paragr.1
	3c	The main diagnoses, therapeutic interventions, and outcomes	P2, Case present, paragr.1
	3d	Conclusion—What is the main "take-away" lesson(s) from this case?	P2, Conclusion, Paragr.1
Introduction	4	One or two paragraphs summarizing why this case is unique (**may include references**)	P3, Introduction, Paragr. 1
Patient Information	5a	De-identified patient specific information	P3, Case report, Paragr. 1
	5b	Primary concerns and symptoms of the patient	P3, Case report, Paragr. 1
	5c	Medical, family, and psycho-social history including relevant genetic information	P3, Case report, Paragr. 2
	5d	Relevant past interventions with outcomes	P4, Case report, Paragr. 3+4
Clinical Findings	6	Describe significant physical examination (PE) and important clinical findings	P4, Case report, Paragr. 4
Timeline	7	Historical and current information from this episode of care organized as a timeline	N/A
Diagnostic Assessment	8a	Diagnostic testing (such as PE, laboratory testing, imaging, surveys).	P4, Case report, Paragr. 4+5
			P5, Case report, Paragr. 8
			P6, Case report, Paragr. 10
	8b	Diagnostic challenges (such as access to testing, financial, or cultural)	P5, Case report, Paragr. 7
Therapeutic Intervention			P6, Case report, Paragr. 9
	8c	Diagnosis (including other diagnoses considered)	P6,Case report, Paragr. 9+10
Follow-up and Outcomes	8d	Prognosis (such as staging in oncology) where applicable	N/A
	9a	Types of therapeutic intervention (such as pharmacologic, surgical, preventive, self-care)	P5, Case report, Paragr. 6
	9b	Administration of therapeutic intervention (such as dosage, strength, duration)	P5, Case report, Paragr. 6
			P6, Case report, Paragr. 10
	9c	Changes in therapeutic intervention (with rationale)	P5, Case report, Paragr. 7
			P6, Case report, Paragr. 10
	10a	Clinician and patient-assessed outcomes (if available)	P6, Case report, Paragr. 10
	10b	Important follow-up diagnostic and other test results	P6, Case report, Paragr. 10
	10c	Intervention adherence and tolerability (How was this assessed?)	P5, Case report, Paragr. 7
	10d	Adverse and unanticipated events	P5, Case report, Paragr. 7
Discussion	11a	A scientific discussion of the strengths AND limitations associated with this case report	P6-8 Discussion
	11b	Discussion of the relevant medical literature **with references**	P6-8 Discussion
	11c	The scientific rationale for any conclusions (including assessment of possible causes)	P7, Discussion, Paragr. 3

		....	P8, Discussion, Paragr. 4
	**11d**	The primary "take-away" lessons of this case report (without references) in a one paragraph conclusion	P8 , Discussion, Paragr. 4
**Patient Perspective**	**12**	The patient should share their perspective in one to two paragraphs on the treatment(s) they received	N/A
**Informed Consent**	**13**	Did the patient give informed consent? Please provide if requested	**Yes** ☒ **No** ☐
