# Supplementary material for: Tuberculosis dissemination in kidney transplant recipient treated with anti-CD40 monoclonal antibody: a case report
Source: BMC Nephrol. 2022 Aug 19;23:290. doi: 10.1186/s12882-022-02916-2 (PMC9388963; doi:10.1186/s12882-022-02916-2)
Supplement: Supplementary file 2 — Additional file 2. TUBERCULOSIS DISSEMINATION IN KIDNEY TRANSPLANT RECIPIENT TREATED WITH ANTI-CD40 MONOCLONAL ANTIBODY: A CASE REPORT. [file 12882_2022_2916_MOESM2_ESM.pdf]

TUBERCULOSIS DISSEMINATION IN KIDNEY TRANSPLANT RECIPIENT TREATED WITH ANTI-CD40 MONOCLONAL ANTIBODY: A CASE REPORT

Kamila Bednarova [1,2], Janka Slatinska[1], Ondrej Fabian[4], Pavel Wohl[5], Emilia Kopecka[6], Ondrej Viklicky [1,3]

[1] Department of Nephrology, Transplant Centre, Institute for Clinical and Experimental Medicine, Prague, Czech Republic.

[2] 1st Medical Faculty, Charles University, Prague, Czech Republic.

[3] Transplant Laboratory, Institute for Clinical and Experimental Medicine, Prague, Czech Republic.

[4] Clinical and Transplant Pathology Centre, Institute for Clinical and Experimental Medicine, Prague, Czech Republic

[5] Department of Hepatogastroenterology, Transplant Centre, Institute for Clinical and Experimental Medicine, Prague, Czech Republic.

[6] Department of Respiratory Medicine, 1st Faculty of Medicine of Charles University and Thomayer Hospital, Prague, Czech Republic.

Correspondence:

Ondrej Viklicky, MD, PhD, Professor of Medicine, Department of Nephrology, Transplant Center,

Institute for Clinical and Experimental Medicine, Videnska 1958/9, 14021 Prague, Czech Republic,

Email: ondrej.viklicky@ikem.cz

Abstract

Background

Tuberculosis (TBC) in solid organ transplant recipients represents a severe complication. The incidence among transplant recipients is higher than in the general population, and the diagnosis and treatment remain challenging. We present a case of active disseminated tuberculosis in a kidney transplant recipient treated with an anti-CD40 monoclonal antibody, who had been previously exposed to an active form of the disease, but latent tuberculosis (LTBI) was repeatedly ruled out prior to transplantation. To the best of our knowledge, no other case has been reported in a patient treated with the anti-CD40 monoclonal antibody.

Case Presentation

A 49-year-old patient, 1.5 years after primary kidney transplantation, presented with vocal cord problems, a dry irritating cough, and a sore throat. A detailed investigation, including a high-resolution chest CT scan, revealed the diagnosis of disseminated tuberculosis. The antituberculosis treatment consisting of rifampicin, isoniazid, pyrazinamide, and ethambutol was started immediately. The patient's condition became complicated by relapsing diarrhoea. The colonoscopy revealed a circular stenosis above Bauhin’s valve. Microscopical findings showed active colitis and vaguely formed collections of epithelioid macrophages without fully developed caseous granulomas and were consistent with the clinical diagnosis of tuberculosis. The antituberculosis treatment was subsequently enhanced by moxifloxacin and led to a great improvement in the patient’s condition.

Conclusion

In this case, false negativity of interferon-γ release assays and possibly higher risk for intracellular infections in patients on costimulatory signal blockers are discussed.

Keywords

case report, costimulation, iscalimab, kidney transplantation, tuberculosis

Background

Tuberculosis represents a life-threatening infection complication in immunosuppressed patients. In solid organ transplant recipients, the risk of tuberculosis is up to 74 times higher compared with the general population. The reactivation of latent tuberculosis in organ transplant recipients within the first twelve months is the most common manifestation (1). Symptoms are rather unspecific, including fevers, night sweats, weight loss, or cough. Moreover, the diagnosis remains challenging and often delayed, leading to a more serious course of the disease. Treatment of post-transplant tuberculosis is demanding due to drug interactions and side effects. Therefore, screening for latent tuberculosis infection prior to transplantation and consequent prophylaxis with isoniazid is essential. Available screening tests for latent tuberculosis, including interferon gamma-release assays (IGRA) and tuberculin skin tests (TST), are limited in patients with chronic kidney disease, as the tests rely on an intact immune response (2). Here, we present a case of post-transplant disseminated tuberculosis, resulting from reactivation of an undiagnosed latent disease.

Case Presentation

A 49-year-old patient suffering from end-stage renal disease caused by IgA nephropathy was admitted to our clinic for one month-long vocal cord problems, a dry, irritating cough, and sore throat 1.5 years after primary kidney transplantation.

The patient has been followed for CKD since 2016 and treated by peritoneal dialysis since 2019. His epidemiological history revealed that the patient was exposed to an active form of tuberculosis in 2007 due to close contact with his mother and sister suffering from the disease at that time. To identify possible latent tuberculosis prior to transplantation, he had been repeatedly tested by interferon-γ release assay (IGRA, QuantiFERON-TB Gold), and a tuberculin skin test with negative results. He had received the BCG vaccination in his childhood. The consultant pulmonologist did not recommend

chemoprophylaxis with isoniazid because the IGRA test was negative and the infection occurred more than 5 years ago.

The patient underwent a successful deceased donor kidney transplant at our centre in July 2019. The immunosuppression was based on a protocol of a randomized multicentre clinical study (NCT03663335) with an inhibitor of the CD40-CD154 costimulatory pathway iscalimab (300mg or 600 mg partially blinded), mycophenolate mofetil (2g at POD, 1g/day at month 2), and steroids (20 mg of prednisone daily tapered down to 5 mg at month 3). In the first week, rabbit antithymocyte globulin (rATG, cumulative dose of 2.9 mg/kg) was used as induction therapy. Protocol biopsies performed in November 2019 and June 2020 revealed no significant fibrosis or tubular atrophy (IF/TA 1) and no glomerulitis. There was only mild fibrointimal thickening of arteries and occasional hyalin insudates within the arteriolar wall. Both biopsies were with no evidence of acute rejection or pathological findings in perirenal adipose tissue. The second biopsy sample showed positive immunofluorescence staining for IgA with minor mesangial hypercellularity. Electron microscopy further confirmed the presence of mesangial deposits. These findings supported the diagnosis of IgA nephropathy recurrence. The patient was infected with COVID-19 in October 2020, showing mild symptoms.

In early February 2021, he suffered from a cough and sore throat. Antibiotic therapy with ampicillin-sulbactam was initiated in the outpatient clinic. Despite the antibiotic treatment, the patient's condition worsened, and he was admitted to our hospital. At presentation, he was febrile of 39 °C, normotensive with a heart rate of 90'. Physical examination revealed bilaterally swollen cervical lymph nodes. Initial laboratory evaluation results displayed the following values: C-reactive protein (CRP) 109 mg/L, creatinine 167 µmol/L, urea 8.9 mmol/L, haemoglobin level (Hb) 109 g/L, a white blood cell count of 11.4×10^9/L, and a platelet count of 410×10^9/L.

Ultrasonography of the abdomen, X-rays of the heart and lungs, paranasal sinuses, and echocardiography were performed for further diagnosis, all without evident abnormalities. The ENT examination excluded other abnormalities, and the diagnosis of acute laryngotracheitis was made.

Empiric antimicrobial therapy was initiated with cefotaxime. Despite a transient decrease in inflammatory parameters, the condition worsened again in the following days with recurrent febrile, cough, and finally intermittent haemoptysis. Therefore, a high-resolution chest CT scan was performed with a surprising finding of multiple small nodules affecting both lungs (Fig. 1F). Due to the current positivity of the aspergillus antigen in the blood, we first thought of aspergillus pneumonia. However, the next day, we received a strongly positive *Mycobacterium tuberculosis* PCR test in the sputum.

The patient was diagnosed with miliary tuberculosis and transferred to the pulmonology department for the treatment of multidrug-resistant tuberculosis. Anti-TB therapy was immediately started with rifampicin, isoniazid, pyrazinamide, and ethambutol. The immunosuppressive therapy with iscalimab and mycophenolate mofetil was discontinued, and tacrolimus-based immunosuppression initiated (target tacrolimus levels 5-7 μg/l).

After a month of antituberculosis treatment, the blood *Mycobacterium tuberculosis* PCR test was repeatedly negative. Despite this, the condition became complicated by relapsing diarrhoea caused by Clostridium difficile colitis and elevation in the liver enzymes. The patient's condition improved after the temporary discontinuation of antituberculosis treatment and the addition of oral vancomycin therapy for Clostridium difficile colitis. After 2.5 months in the hospital, the patient was discharged with triple antituberculosis therapy.

Next, the diarrhoea relapsed in the following month, and the patient was readmitted. Clostridium difficile toxin and antigen were both negative. Endoscopic findings in the upper gastrointestinal tract were unremarkable, apart from a small fundic gland polyp in the stomach and a polyp in the duodenal bulb. The colonoscopy revealed circular stenosis above Bauhin's valve (Fig. 1E) and mild oedema in the rectum. Multiple biopsies were obtained. In histological findings in the upper gastrointestinal tract, we found only a single isolated collection of strongly periodic acid Shiff (PAS) positive foamy macrophages in duodenal mucosa. In the region of stenosis, microscopy revealed severe active colitis with disrupted mucosal architecture, foci of cryptitis, crypt abscesses, and the presence of deep ulcerations (Fig. 1A-

B). In the adjacent mucosa, there were loosely cohesive collections of macrophages with epithelioid morphology. However, no fully developed granulomas with caseous necrosis were detected. Other regions of the large bowel showed only mild chronic colitis of a non-specific morphology. Ziehl-Nielsen stain for acid-fast bacilli was negative, as well as PCR detection of mycobacterium species DNA from bioptic tissue. PAS staining was negative for mycotic or parasitic structures. Neither viral inclusions nor vasculitis could be seen. Because of a deterioration in kidney graft function, a graft biopsy was performed. Histology revealed normocellular glomeruli with no evidence of glomerulitis or transplant glomerulopathy. There was no significant fibrosis or inflammation within the interstitium. Renal vessels showed only minor fibrointimal thickening of arteries and occasional hyalin insudates within the arteriolar wall. There were no signs of intimal arteritis, microvascular inflammation, or other signs suggestive of acute rejection. Surprisingly, within the perirenal fibrous tissue, the well-defined epithelioid granuloma was identified, showing no evidence of adjacent birefringent material or foreign body-type giant cell reaction. Despite no caseous necrosis being present within the granuloma, this finding is well in keeping with mycobacterial infection, further supporting the previously established clinical diagnosis of TBC (Table 1).

The microscopical findings supported the clinical diagnosis of mycobacterial infection. Vaguely formed collections of epithelioid macrophages without fully developed caseous granulomas could suggest atypical mycobacteriosis, but they were also in keeping with the diagnosis of tuberculosis, since the morphology of the inflammatory response may have been modified by immunosuppression. Regarding the collection of PAS+ foamy macrophages in the duodenum, Whipple's and Crohn's diseases were discussed as broader differential diagnoses.

On this basis, a gastroscopy and colonoscopy were repeated at a two-month interval. Macroscopically, the findings in the upper gastrointestinal tract were similar, now with almost normal duodenal findings. The colonoscopy showed pancolitis with persistent stenosis in the ascending colon. The morphological assessment revealed resolving colitis with healing ulcerations and adjacent fibrosis in the region of endoscopically described stenosis and in the terminal ileum mild chronic ileitis with a

single isolated epithelioid microgranuloma was present (Fig. 1C-D). These findings corresponded with the clinical diagnosis of protracted mycobacterial infection, and Crohn's and Whipple's diseases were excluded. The diagnosis of disseminated tuberculosis was thus established; the antituberculosis therapy at that time with rifampicin and isoniazid was enhanced by moxifloxacin for 4 months until December 2021. In November 2021, a control colonoscopy was performed and further improvements in macroscopy and morphology were noticed. Kidney graft function remained stable (eGFR 40 ml/min/1,73m^2), recent immunosuppression is based on tacrolimus and steroids. Concerning the improving colonoscopy results and relapsing Clostridium difficile infections, the antituberculosis therapy was reduced to isoniazid and rifampicin.

Discussion and Conclusion

Tuberculosis still represents a rare disease in Central Europe. The incidence in 2020 is 3.4/100,000 in the Czech Republic (3). The prevalence of TBC is around 1.2-6.4% in developed countries, while in endemic countries is 10-15% (1, 4). As we presented above, tuberculosis in immunosuppressed patients may be presented either as the reactivation of latent or *de-novo* disease (1, 4) and its diagnosis can be challenging. Finally, we detected miliary tuberculosis dissemination in the lungs, granulomas were present both in the intestine and in the kidney allograft. Our case pointed out two important issues. At first, there was false negativity of interferon-γ release assay and TST tests despite referred patient's close contact with tuberculosis more than a decade ago, and secondly, the patient had experienced long-lasting inhibition of costimulatory signal of T-cell activation by iscalimab and T-cell depletive induction immunosuppression.

The sensitivity of interferon-γ release assays has been reported to be as high as 92% in patients with active disease. However, for LTBI the only gold standard is the later development of an active form of the disease and, therefore, it can be anticipated that sensitivity of interferon-γ release assays is far lower in CKD patients with an impaired immune system which is further aggravated by immunosuppression later after transplantation (5, 6). A recent prospective study from Taiwan analysed

425 patients with latent TBC in kidney transplant candidates on the waiting list and after kidney transplantation (7). False negativity of interferon-γ release assay before transplantation was associated with a longer interval since *Mycobacterium tuberculosis* infection after transplantation. Positive interferon-γ release assay conversions, in particular, were found in 20% of cases two years after transplantation. Among other risk factors for LTBI, older age, absence of BCG vaccination, and positive donor-specific antibodies were described.

CKD patients on immunosuppression represent a high-risk cohort for LTBI progression to active disease because T cells and antigen-presenting cells represent a critical tool in eliminating intracellular pathogens. As a result, the National Institute for Health and Care Excellence (NICE), recently recommended preventive treatment in all immunocompromised patients regardless of a negative interferon-γ release assay or Mantoux test (8), implying that the potential benefit of preventive treatment outweighs the potential harm in this specific cohort. In comparison, in the new National Tuberculosis Controllers Association (NTCA) recommendations, the immunosuppressed patients, with a recent (<2 years) close contact with pulmonary TB disease, should be treated for LTBI regardless of their screening test. Such a recommendation does not take into account a situation with a long history of close contact (9). Similarly, our local 2019 guideline on management of latent tuberculosis in kidney transplant recipients warns of the risk of a false-negative test in immunocompromised patients but does not explicitly recommend treatment in patients with a negative test result and a history of contact with active TB (10). Undoubtedly, our patient should have received tuberculosis preventive therapy along with the initiated posttransplant immunosuppression. The decision not to do so was based on again relatively vague previous WHO recommendations (11, 12), repeated negative both interferon-γ release and tuberculosis skin tests, and finally a distant history of the patient's contact with tuberculosis.

Moreover, in this case, the patient was put at even higher risk by selecting immunosuppression that affects T-cell response differently than standard calcineurin inhibitors. Iscalimab, a costimulatory signal

blocker, is a fully human nondepleting anti-CD40 monoclonal antibody that blocks CD154 binding to CD40 and downstream pathway activation and has been tested in early phases of clinical development in various autoimmune disorders and kidney transplantation (13). *Mycobacterium tuberculosis* has also been shown to modulate costimulatory molecules by reducing the CD40 expression to further invade the host (14). Interaction between the CD40 receptor and CD40L on activated T cells upregulates the production of IL-12, reactive oxygen species and IFN-γ production. Thus, it plays a crucial role in eliciting cell-mediated immunity important in the defence against intracellular infection (15, 16). As a result, transplant recipients treated with costimulatory signal blockers are likely to be at higher risk of tuberculosis than those on calcineurin inhibitors. Tuberculosis is a rare infection in most western countries, and costimulatory signal blockers as maintenance immunosuppression have been used just in a small percentage of transplant recipients. Therefore, it is highly unlikely to get any other safety signal than that from clinical trials. Interestingly, in the 3-year follow-up of the BENEFIT-EXT trial, tuberculosis was reported in 6 out of 223 patients treated with belatacept, a selective costimulation blocker preventing T-cell activation, while tuberculosis was not reported among the 100 patients treated with cyclosporine (17). However, our experience from a single case does not allow us to draw any meaningful conclusion and cannot demonstrate any association between iscalimab and mycobacterium tuberculosis infection.

To the best of our knowledge, this is the first clinical report of reactivation of latent tuberculosis in a patient treated with the anti-CD40 monoclonal antibody after kidney transplantation.

Tables

Table 1. Affected organs by tuberculosis

AFFECTED ORGAN	ORGAN DAMAGE<br>Macroscopy findings	Microscopy findings
**LUNGS**	multiple small nodules affecting both lungs	N.D.
**INTESTINE**<br>**Ascending colon, caecum**	circular stenosis above Bauhin's valve	severe active colitis, foci of cryptitis, crypt abscesses, deep ulcerations, macrophages with epithelioid morphology in the mucosa
**Transverse and descending colon**	normal	mild chronic colitis of a non-specific morphology, in second biopsy pseudomembrane formation (CDI)
**Rectum + anal canal**	haemorrhoids	mild chronic colitis of a non-specific morphology,
**KIDNEY**	N.D.	epithelioid granuloma in perirenal adipose tissue.

N.D.: not done

Figure legends:

Fig. 1. Tuberculosis manifestation.

A-B: Bioptic samples from the stenotic region of the large bowel (A) and from the duodenum (B). **A**: The architecture of the mucosa is impaired, and the crypts are distorted and cystically dilated. In the lamina propria, there is a severe chronic active inflammation with crypt abscess and ulceration. In deeper parts of the bowel mucosa and submucosa, there is a vaguely formed collection of epithelioid macrophages surrounded by a chronic inflammatory infiltrate (haematoxylin and eosin, magnification 100x); **B:** In the lamina propria, there is a collection of foamy macrophages strongly positive in the periodic acid-Shiff stain. The rest of the mucosa is devoid of any inflammation and the architecture is intact (periodic acid-Shiff, 200x); **C-D:** Repeated biopsy after two-months internal. Bioptic samples from the inflamed colonic mucosa (C) and terminal ileum (D).

C: There is chronic active colitis with largely preserved mucosal architecture. The activity of the inflammation is superficially localized, with neutrophils infiltrating surface epithelium. In the centre of the picture, there is a small mucosal defect with incipient exudation of fibrin (haematoxylin and eosin, 200x); **D**: In the centre of the picture, there is an isolated mucosal collection of epithelioid macrophages (epithelioid microgranuloma). The surrounding mucosa is slightly oedematous and hyperaemic (haematoxylin and eosin, 200x); **E**: Circular stenosis above Bauhin's valve; **F**: Chest computed tomography (CT). Axial thin-section unenhanced CT image revealing uniformly distributed miliary deposits in both lungs. Equipment used for microscopy images: Microscope- Olympus BX41; camera Canon EOS 700D; Acquisition software- QuickPhoto Camera 3.2. No enhancement of the images was performed. The images were acquired at a resolution of 300 DPI.

Abbreviations

ATG: Antithymocyte globulin

CKD: Chronic kidney disease

ENT: ears-nose-throat

IGRA: Interferon gamma-release assay

LTBI: Latent tuberculosis infection

POD: Postoperative day

TBC: Tuberculosis

TST: Tuberculin skin tests

Declarations

Ethics approval and consent to participate

Not applicable.

Consent for publication

Written informed consent was obtained from the patient for publication of this case report and any accompanying images. All patient data was anonymized. A copy of the written consent is available for review. This manuscript does not require other. The research was conducted ethically in accordance with the World Health Association Declaration of Helsinki.

Availability of data and material

The datasets used and analysed during the current study are available from the corresponding author on reasonable request.

Competing interests

The authors have no conflicts of interest to declare.

Funding

Supported by Ministry of Health, Czech Republic - conceptual development of research organization („Institute for Clinical and Experimental Medicine – IKEM, IN 00023001“)

The funding body played no role in study design, data collection, analysis, interpretation, or manuscript writing.

Authors' contributions

KB and JS were equally involved in the writing of the case report and discussion of the manuscript. OV supervised and corrected the manuscript. OF was involved in the interpretation of pathology findings. PW was involved in patient management and editing of the manuscript. EK participated in the clinical care of the patient. All authors read and approved the manuscript.

Acknowledgements

Not applicable

References:

1. Anand M, Nayyar E, Concepcion B, Salani M, Schaefer H. Tuberculosis in kidney transplant recipients: A case series. World J Transplant. 2017;7(3):213-21.
2. Bumbacea D, Arend SM, Eyuboglu F, Fishman JA, Goletti D, Ison MG, et al. The risk of tuberculosis in transplant candidates and recipients: a TBNET consensus statement. Eur Respir J. 2012;40(4):990-1013.
3. UZIS Health Statistics. Basic Overview of Tuberculosis Epidemiology in the Czech Republic in 2020. 2021. ISSN: 1210-8685, (0862-5670). Available online: https://www.uzis.cz/res/f/008358/tbc2020-en.pdf.
4. Sasi S, Varghese MK, Nair AP, Hashim S, Al Maslamani M. Tuberculosis in an Allogeneic Transplant Kidney: A Rare Case Report and Review of Literature. Cureus. 2020;12(11):e11661.
5. Diel R, Loddenkemper R, Niemann S, Meywald-Walter K, Nienhaus A. Negative and positive predictive value of a whole-blood interferon-γ release assay for developing active tuberculosis: an update. Am J Respir Crit Care Med. 2011;183(1):88-95.
6. Hornum M, Mortensen KL, Kamper AL, Andersen AB. Limitations of the QuantiFERON-TB Gold test in detecting Mycobacterium tuberculosis infection in immunocompromised patients. Eur J Intern Med. 2008;19(2):137-9.
7. Shu CC, Tsai MK, Lin SW, Wang JY, Yu CJ, Lee CY. Latent Tuberculosis Infection Increases in Kidney Transplantation Recipients Compared With Transplantation Candidates: A Neglected Perspective in Tuberculosis Control. Clin Infect Dis. 2020;71(4):914-23.
8. National Institute for Health and Care Excellence (NICE). Tuberculosis. NICE guideline [NG33] [12 September 2019:[Available from: www.nice.org.uk/guidance/ng33.

9. TB Controllers. A GUIDE FOR HEALTH CARE PROVIDERS AND PUBLIC HEALTH PROGRAMS - Testing and Treatment of Latent Tuberculosis Infection in the United States: Clinical Recommendations.http://www.tbcontrollers.org/docs/resources/tb-infection/LTBI_Clinical_Recommendations_Version_002052021.pdf (Accessed on March 22, 2022).
10. Polcova V, Vasakova M, Valentova-Bartakova L. Tuberkulóza (TB) a latentní tuberkulózní infekce u pacientů před a po transplantaci solidních orgánů či hematopoetických kmenových buněk (Doporučený postup diagnostiky a léčby). http:/www.pneumologie.cz/guidelines2016. p. Kap. 4.5.
11. World Health Organization. (2018). Latent tuberculosis infection: updated and consolidated guidelines for programmatic management; Licence: CC BY-NC-SA 3.0 IGO; World Health Organization: Geneva, Switzerland, 2018. Available online: https://apps.who.int/iris/handle/10665/260233. (accessed on 20 February 2022)
12. World Health Organization. WHO Consolidated Guidelines on Tuberculosis. In Module 1: Prevention—Tuberculosis Preventive Treatment; Licence: CC BY-NC-SA 3.0 IGO; World Health Organization: Geneva, Switzerland, 2020. Available online: https://apps.who.int/iris/bitstream/handle/10665/331170/9789240001503-eng.pdf?sequence=1&isAllowed=y (accessed on 20 February 2022).

13. Espié P, He Y, Koo P, Sickert D, Dupuy C, Chokoté E, et al. First-in-human clinical trial to assess pharmacokinetics, pharmacodynamics, safety, and tolerability of iscalimab, an anti-CD40 monoclonal antibody. Am J Transplant. 2020;20(2):463-73.

14. Khan N, Gowthaman U, Pahari S, Agrewala JN. Manipulation of costimulatory molecules by intracellular pathogens: veni, vidi, vici!! PLoS Pathog. 2012;8(6):e1002676.
15. Chauhan P, Dandapat J, Sarkar A, Saha B. March of Mycobacterium: miRNAs intercept host cell CD40 signalling. Clin Transl Immunology. 2020;9(10):e1179.
16. Sia JK, Bizzell E, Madan-Lala R, Rengarajan J. Engaging the CD40-CD40L pathway augments T-helper cell responses and improves control of Mycobacterium tuberculosis infection. PLoS Pathog. 2017;13(8):e1006530.
17. Pestana JO, Grinyo JM, Vanrenterghem Y, Becker T, Campistol JM, Florman S, et al. Three-year outcomes from BENEFIT-EXT: a phase III study of belatacept versus cyclosporine in recipients of extended criteria donor kidneys. Am J Transplant. 2012;12(3):630-9.
