# Supplementary material for: Tuberculosis dissemination in kidney transplant recipient treated with anti-CD40 monoclonal antibody: a case report
Source: BMC Nephrol. 2022 Aug 19;23:290. doi: 10.1186/s12882-022-02916-2 (PMC9388963; doi:10.1186/s12882-022-02916-2)
Supplement: Supplementary file 3 — Additional file3 Reviewer reports. [file 12882_2022_2916_MOESM3_ESM.docx]

July 29^th^, 2022

# To:

Dr. Sarah Gray

BMC Nephrology

Dear Dr. Sarah Gray,

Thank you for reviewing our manuscript entitled “Tuberculosis dissemination in kidney transplant recipient treated with anti-CD40 monoclonal antibody: A case report”, as well as for the invitation to resubmit a revised version of our manuscript.

We have modified the manuscript according to the editors’ and referees’ suggestions. Please see the point-by-point responses to the editors’ and reviewers’ comments as listed below. We are grateful for the review of the revised manuscript.

On behalf of authors,

Yours sincerely,

Ondrej Viklicky, MD, PhD

Professor of Medicine

Department of Nephrology, Transplant Center

Institute for Clinical and Experimental Medicine (IKEM), Prague

**Editor Comments:**

**1. Please note that all manuscripts must contain all the following sections under the heading 'Declarations'. The Declarations should follow the Abbreviations, and be before the References.
Please do not deviate from the following order, or naming of titles.**

Ethics approval and consent to participate
Consent for publication
Availability of data and material
Competing interests
Funding
Authors' contributions
Acknowledgements

**Reply:** Thank you for this suggestion, and we apologize for this inaccuracy, which has been corrected.  (p. 12; line 26-54; p. 13; line 1-32)

**2. In order to be in line with journal requirements, please include the following headings within your manuscript: Background, Case Presentation, Discussion and Conclusion (where Discussion and Conclusion are one section).**

**Reply:** We appreciate your suggestion, we have included requested headings within the manuscript. (p. 3; line 1), (p. 3; line 35), (p. 7; line 23)

**3. - In the Methods, please specify the type of equipment and acquisition software used to capture the microscopy images included in your study.
- You should state the measured resolution at which an image was acquired and any downstream processing or averaging that enhances the resolution of the image.
- Please also ensure that a scale bar is included with every microscopy image.**

**Reply:** Thank you for this comment, and we apologize for missing information. We have provided further details on the type of equipment and acquisition software in the manuscript. We have added the scale bars to all microscopy images. (p. 11; line 38-48) **4. In the Funding section, please also describe the role of the funding body in the design of the study and collection, analysis, and interpretation of data and in writing the manuscript. If they had no role in the design of the study and collection, analysis, and interpretation of data and in writing the manuscript please clearly state this.**

**Reply**: Thank you for this comment. We have made a clear statement in the manuscript. (p. 13; line 11-14)

**Reviewer reports:**

**Reviewer 2**:

**I am happy with the revision and I think that the authors managed to significantly improve the overall quality of the manuscript.
If possible, I would suggest further language polishing.**

**Reply:** We thank you for this suggestion. Further proofreading of our manuscript has been done.

**Reviewer 3:**

**No further recommendation.**
